# Oxidative stress and cardiovascular risk related to urinary metal(loid) levels in a pediatric population

**DOI:** 10.1007/s10653-026-03190-z

**Published:** 2026-04-22

**Authors:** Manolo Ortega-Romero, Elodia Rojas Lima, Juan Carlos Rubio-Gutiérrez, Juana Narváez Morales, Ixtlitzin Yaocihuatl Bravo Carvajal, Mariela Esparza García, Miguel Ángel Mejia, Juan Alfredo Tamayo y Orozco, Pablo Mendez-Hernández, Olivier C. Barbier, Mara Medeiros, Octavio Gamaliel Aztatzi-Aguilar

**Affiliations:** 1 Unidad de Investigación en Nefrología y Metabolismo Mineral Óseo, Hospital Infantil de México Federico Gómez, México, CDMX México; 2 Departamento de Toxicología, Centro de Investigación y de Estudios Avanzados, Mexico, CDMX México; 3 Fundación Franco-Mexicana para la Medicina, I.A.P, México, CDMX México; 4 Accessalud, México, CDMX México; 5 Departamento de Calidad y Educación en Salud, Secretaría de Salud de Tlaxcala, Tlaxcala, México; 6 Facultad de Ciencias de la Salud, Universidad Autónoma de Tlaxcala, Tlaxcala, México; 7 Departamento de Farmacología, Facultad de Medicina, Universidad Nacional Autónoma de México, México, CDMX México; 8 Universidad Nacional Autónoma de México, México, CDMX México

**Keywords:** Oxidative stress, Cardiovascular risk, Metal(loid)s, Pediatric population

## Abstract

Cardiovascular diseases are the leading cause of death, and reducing them requires identifying risk factors, among which exposure to environmental pollutants is well documented. Metal(loid) exposure is related to oxidative stress (OxS), in which oxygen radicals react with biomolecules, thus altering the cardiovascular system. This study aimed to identify cardiovascular risk factors and their relationship with OxS biomarkers. A cross-sectional study (CINVESTAV-063-2020/HIM-2019-025) was conducted in an apparently healthy pediatric population (N = 359). Metal(loid)s were measured by ICP-MS, and enzymatic and colorimetric techniques were applied for OxS determination. Descriptive and bivariate analyses were performed. A weighted quantile sum (WQS) approach was used to examine the mixture effect and identify the components associated with a health outcome. Finally, spatial autocorrelation was estimated for metal(loid)s and environmental sources. The median age of the participants was 14 years, 30% were overweight or obese, and cardiovascular risk was 32.6%. Median metal(loid) concentrations (ng/mL) were 34.5 for arsenic, 27.2 for copper, 0.837 for manganese, and 7.45 for vanadium. There was an association between the multi-OxS biomarkers and cardiovascular risk (OR: 0.4291, 95% CI: 0.281–0.577). However, there was no relationship between the multi-metal(loid)s and cardiovascular risk, but there were significant correlations between the OxS biomarkers and urinary metal(loid)s. Spatial autocorrelation was observed for vanadium and arsenic. OxS biomarkers were associated with cardiovascular risk and some related factors. Furthermore, the presence of metal(loid)s is involved in redox imbalance, which appears to increase cardiovascular risk, and the spatial autocorrelation observed suggests exposure to vanadium and arsenic.

## Introduction

Cardiovascular diseases (CVDs) are among the leading causes of death and disability worldwide (Di Cesare et al., 2024). In addition to an increase in CVDs in the population (Qasim et al., 2016), approximately 18 million deaths from this cause were reported in 2017, which corresponds to 330 million years of life lost (Yang et al., 2020). Therefore, identifying risk and modifiable factors is of the utmost urgency in order to reduce the adverse impact of these diseases on human health (Francula-Zaninovic & Nola, 2018). For example, the Framingham heart studies (Levy et al., 1990; Mosca et al., 2007) have identified and summarized risk factors with pathogenic implications for CVDs, including smoking, hypertension, dyslipidemia, diabetes, etc. (Brunelli et al., 2017). On the other hand, exposure to toxic metal(loid)s has become a public health concern due to their potentially harmful effects (Jimenez-Cordova et al., 2018; Ortega-Romero et al., 2023), including their cardiovascular outcomes (Mendez et al., 2016), although the results are not conclusive. It is also unclear whether there is a detrimental association with CVD at exposure levels below those considered safe (Chowdhury et al., 2018).

Experimental and clinical studies suggest a relationship between CVDs and biomarkers of oxidative stress (OxS), which occurs when there is an imbalance between antioxidant and pro-oxidant activities (Tursi Ríspoli1 et al., 2013). When reactive oxygen species (ROS) are produced in excess, they react with carbohydrates, lipids, DNA, and proteins, altering their structures and functions (Qasim et al., 2016). An increased production of ROS and a reduced antioxidant metabolism have been implicated in the pathogenesis of CVDs, including hypertension, atherosclerosis, cardiac hypertrophy, heart failure, restenosis, and related pathologies such as diabetes mellitus (Yung et al., 2006). In addition, OxS stimulates myocardial growth, matrix remodeling, and cellular dysfunction, which involves the activation of several downstream signaling pathways. ROS induce apoptosis, which is another important contributor to remodeling and dysfunction, cause ruptures in the myocardium, are involved in the regulation of the expression of inflammatory mediators, which facilitate cardiac remodeling, and directly influence contractile function by modifying the proteins involved in excitation–contraction coupling (Dubois-Deruy et al., 2020). However, the utility of OxS biomarkers for clinical prognosis and their use to estimate cardiovascular risk have been poorly investigated. Several biomarkers can be analyzed to investigate OxS, including ROS and the end products of lipid-aldehyde malondialdehyde (MDA) and 4-hydroxy-2-nonenal (4HNE) (Usberti et al., 2002). It was recently found that CVD patients have higher plasma MDA levels, which were an independent discriminator of CVD (De Tursi Rispoli et al., 2013). However, due to the very short half-life of ROS, it is impossible to measure them directly, and thus it is necessary to obtain measurements of antioxidant scavenging enzymes, such as erythrocyte superoxide dismutase (SOD) and catalase (CAT), or lipid and protein oxidation by-products to assess OxS (Vaziri et al., 2003). The growing evidence suggests that an increase in OxS is related to chronic inflammation, and one of the most important consequences of this is accelerated atherosclerosis (Ece et al., 2006). Metal(loid)s can induce OxS by generating and promoting ROS, including superoxide radicals, hydrogen peroxide, and nitric oxide (Solenkova et al., 2014). In addition, it has been shown that metal(loids)s increase lipid peroxidation, or the oxidative modification of low-density lipoprotein, which is related to the development of atherosclerosis (Srivastava et al., 2009). For example, cadmium (Cd) can damage vascular tissues, induce endothelial dysfunction, and promote atherosclerosis, while lead (Pb) induces protein and nucleic acid degradation and lipid peroxidation (Peters et al., 2012). On the other hand, a deficiency of essential metal(loid)s can impair immune function and, through a series of interrelated processes, promote CVD, including an uncontrolled release of inflammatory cytokines, kidney damage, and damage to the central nervous system (Yang et al., 2020).

The different risk factors that predispose to CVD, such as hypercholesterolemia, diabetes, obesity, hypertension, and aging, lead to dysfunction and vascular disease, partly through OxS, which therefore becomes a common denominator of vascular disease induced by risk factors in vascular aging, where two characteristic features related to ROS have been defined: endothelial dysfunction and central arterial stiffness (Munzel et al., 2017). Considering the above, the present study aimed to identify environmental cardiovascular risk factors and determine their relationship with OxS biomarkers, as well as to estimate the spatial autocorrelation of metal(loid)s and environmental exposure sources.

## Material and Methods

### Study design

A sample of 359 individuals between 11 and 18 years of age was randomly selected from the CINVESTAV-HIMFG biobank; with the protocol being approved by ethic committee (CINVESTAV-06312020/HIM-2019-025). A cross-sectional study was conducted in accordance with the Declaration of Helsinki, which included obtaining signed letters of assent and informed consent. The participants underwent a medical review, and the socioenvironmental questionnaire included household characteristics, chronic-degenerative diseases, alcohol and tobacco consumption, personal history, pathological history, education, etc. Blood pressure was measured in duplicate with a sphygmomanometer. Weight and height were measured using a clinical scale and a stadiometer, respectively, while body mass index (BMI) was calculated with the STAT Growth Charts TM application 3.2 from the Center for Disease Control (CDC).

### Study definitions

The following risk factors were identified based on the integrated guidelines for cardiovascular health and risk reduction in children and adolescents [19], where individuals considered to be at risk are those with obesity or overweight, hypertension, and at least one more characteristic. A body mass index between the 85th and 95th percentiles indicate overweight, and a value greater than the 95th percentile indicates obesity, according to the parameters of the World Health Organization (WHO). Age, sex, and height percentiles were considered for the diagnosis and classification of arterial hypertension. In this case, systolic or diastolic blood pressure between the 90th and 95th percentiles was considered indicative of prehypertension, while arterial hypertension exceeded the 95th percentile (Flynn et al., 2017). A sedentary lifestyle was defined as the absence of any physical activity (any activity that accelerated the heartbeat and left the individual breathless on some occasions) for at least one hour a day, three times a week. Bad eating habits were defined as a diet without enough fruit or vegetables, including fried food, desserts, or junk food, two or more times a day. Smokers were defined as those who smoked cigarettes in any amount or frequency (Expert Panel on Integrated Guidelines for Cardiovascular et al., 2011).

### Sample collection

Urine samples were collected from the first morning urine in sterile polypropylene containers, and centrifuged at 1000 g for 10 min, and aliquots were subsequently separated in 1.5 mL eppendorf tubes. Blood samples were collected by peripheral venous puncture; 5 mL of blood were obtained in a dry tube (BD) and another 5 mL in an EDTA (BD) tube. All samples were stored at − 70 ºC.

### Determination of metal(loid)s in urine

Urine concentrations of the metal(loid)s arsenic (As), copper (Cu), manganese (Mn), and vanadium (V) were measured using a Perkin Elmer NexION 300D ICP-MS according to the procedure described by the Laboratory of Research and Service in Toxicology (LISTO) of Cinvestav, accredited by the Mexican accreditation entity (No. INV-0007-013/19). Calibration curves were prepared at different multi-element concentrations (0, 0.1, 0.5, 1.5, 10, and 100 ng/mL). Urine samples were diluted 1:10 in 0.16% ultrapure nitric acid (HNO_3_). Readings were taken from water blanks, air, and the calibration solutions, starting with the lowest concentrations, followed by reference samples, and finally the diluted samples; all measurements were performed in duplicate. The results are expressed in ng/mL. Six certified reference samples of trace elements in urine at different concentrations were obtained from the INSPQ (INSPQ/toxicologie QM-U-Q1705, urine) and used to validate the analysis of the concentrations of inorganic elements in urine. The analysis indicated variation coefficients of less than 10% and recovery percentages of 80–120%. Urinary metal(loid) concentration was normalized to urinary density.

### Determination of oxidative stress biomarkers in plasma

All samples were thawed and immediately analyzed for a total of one freeze–thaw cycle before use.

*Malondialdehyde* (MDA); An aliquot of 25 μL of plasma was added to 185 μL of a solution of 10 mM 1-methyl-2 phenylindole in a mixture of acetonitrile/methanol (3:1). The reaction was then initiated by adding 40 μL of 37% hydrochloric acid. The mixture was incubated at 45 °C for 40 min, samples were centrifuged at 9000 rpm, and supernatants were placed in 96-well plates and measured at 586 nm. Absorbance was read using an Infinite® 200 PRO plate reader (TECAN). A standard curve was processed in the same way and absorbances were interpolated (Gerard-Monnier et al., 1998).

*Myeloperoxidase* (MPO); Peroxidase activity was measured as follows: a 10 μL plasma sample was combined with 90 μL of a solution mix that contained 210 μL of TMB solution (1.6 mM TMB), 1.7 mL of 100 mM sodium phosphate buffer at pH 7.4, and 84 μL of 0.3 mM H_2_O_2_ (Sigma). The samples were incubated at 37 °C for 3 min. The reaction was stopped by adding 150 μL of 0.4 M cold acetate buffer pH 3, and absorption was measured at 590 nm using an Infinite® 200 PRO plate reader (TECAN) (Suzuki et al., 1983).

*Methylglyoxal* (MGO); This biomarker was determined following the 2, 4-dinitrophenylhydrazine (DNPH) alpha-keto acid method reported by Kwok et al. coupled with a microplate assay. Briefly, 20 μL of plasma samples were plated and mixed with 100 μL of DNPH (0.9 mM in 1N HCl) and incubated at 37 °C for 10 min. After incubation, the reaction was stopped by adding 100 μL of NaOH (1.5 N). The colored product was read at 540 nm in an Infinite® 200 PRO plate reader (TECAN). An MGO standard curve was processed in the same way and absorbances were interpolated.

*Arginase*; For this biomarker, the microplate method was adapted from Corraliza et al. (Corraliza et al., 1994). The plasma sample was diluted 1:10 with water and incubated at 55 °C for 10 min. Aliquots of 50 μL of L-Arginine were used to obtain a final concentration of 0.25 M in a final volume of 100 μL, which was then incubated for 1 h at 37 °C. After incubation, a reaction mix of 150 μL was prepared in a proportion of 1:15, composed of 9% iso-nitroso-propiophenone dissolved in ethanol and an acid mixture (H_2_SO_4_, H_3_PO_4_, H_2_O; 1:3:7 v/v). The final reaction was heated at 100 °C for 45 min. The samples were placed in ice and kept upright for 10 min in the dark before being read at 540 nm; absorbance was read using an Infinite® 200 PRO plate reader (TECAN).

*Catalase* (CAT); Catalase activity was determined by the oxidation of Co II to Co III in the presence of H_2_O_2_, adapted to the microplate method. For this, a 20 μL plasma sample was mixed with 40 μL of 10 mM H_2_O_2_ and incubated at 37ºC for 2 min. Subsequently, 240 μL of the reaction mix (69.6 mM Co(NO_3_)_2_; 16.35 mM Graham solution (NaPO_3_)_6_; 1.07 M NaHCO_3_; mixed in that order in a proportion of 1:1:18 v:v:v) were added and immediately read at 440 nm using an Infinite® 200 PRO plate reader (TECAN); catalase activity was calculated with respect to the standard (10 mM hydrogen peroxide).

*Non-esterified fatty acids* (NEFAs); The following method was adapted from Duncombe (1964) and Chilliard et al. (1984) to a microassay. Briefly, plasma samples (50 µl) were treated with 200 µl of an extraction solution (heptane, chloroform, and methanol; 20:20:1, v:v:v), shaken, and centrifuged at 1000 rpm for 2 min to form a micelle, which was removed, and the supernatant was mixed with a NaCl-saturated aqueous 0.5 M cupric nitrate [Cu(NO_3_)_2_ * _3_H_2_O] solution and 1 M triethanolamine at pH 8.1. A new micelle was then formed after shaking and centrifuging at 1000 rpm for 2 min. The micelle was extracted and sodium diethyldithiocarbamate (0.2% dissolved in ethanol) was then added to the supernatant to form a yellow complex with maximum absorbance at 450 nm. Absorbance was read using an Infinite® 200 PRO plate reader (TECAN).

*Glutathione-S-Transferases* (GSTs); GST activity was measured according to the method by Habig (Habig et al., 1974) using chlorodinitrobenzene (CDNB) (Aldrich, Steinheim, Germany) as a substrate and reduced glutathione (Sigma). The formation of the GST-CDNB conjugate was monitored every minute for 10 min by measuring the change in absorbance at 340 nm, which was recorded using an Infinite® 200 PRO plate reader (TECAN). The concentration of GSTs is expressed in nmol/min/mg protein.

*Gamma-glutamyl transferase* (γGT); The γGT assay was performed using the buffer tris-gly/gly pH 8.2 (Trizma-HCl 120 mM, MgCl 12 mM, and Gly/Gly 90 mM) as a substrate and L-γ-glutamyl-p-nitroanilide 10 mM. Briefly, 10 μL of plasma were mixed with 170 μL of the buffer tris-gly/gly pH 8.2 and 20 μL of substrate. The mixture was shaken and 9 readings were obtained at 405 nm during 16 min. Total plasma protein concentration was measured according to the Bradford method, and absorbance was determined at 590 nm using an Infinite® 200 PRO plate reader (TECAN). Bovine serum albumin (BSA, Sigma) was used as a standard.

*Advanced oxidation protein products* (AOPPs); AOPPs were quantified according to the method described by Witko-Sarsat et al. (Witko-Sarsat et al., 1998). For this, 20 μL of plasma were mixed with 140 μL of PBS1X and 20 µL of potassium iodide (KI). The mixture was shaken for 2 min, and 40 µL of pure acetic acid were subsequently added and the mixture was shaken again for 2 min and read at 340 nm in an Infinite® 200 PRO plate reader (TECAN). A standard curve was generated with chloramine-T, and the results are expressed as micromoles of chloramine-T per milligram of total protein.

### Spatial autocorrelation of exposure and environmental sources

Spatial autocorrelation (SA) measures the degree to which a geographic variable is correlated with itself at two different points or zones within a study area. It measures the similarity of the variable within a given area. For this analysis, the fixed distance band was estimated from a neighborhood count, that is, the minimum, maximum, and average distances to the specified Nth nearest neighbor for a set of entities. Each entity had at least eight nearest neighbors to allow for a robust analysis.

The data used to perform the SA included the subject’s home address, data from the federal database of industry substance records from 2020 from the “Registro de Emisiones y Transferencia de Contaminantes (RETC)”, and data from industries registered in the Directorio Estadístico Nacional de Unidades Económicas (DENUE). Industries were classified according to manufacturing activity.

To calculate incremental spatial autocorrelation, Global Moran’s I (Chen, 2023) was used for a series of increasing distances, measuring the intensity of clustering or grouping of spatial data for each distance. The intensity of clustering is determined by the z-score and the returned *p*-value.

### Statistical analysis

Exploratory and descriptive analyses were performed to evaluate the quality of the data and the distribution of the variables of interest. All OxS biomarker and urine metal(oid) concentrations had non-normal residuals and were therefore log_2_ transformed. Mann–Whitney U and Spearman correlation tests were performed to assess the relationship among the OxS biomarkers, the urine metal(oid) concentrations, and the covariates of child age (years), sex, BMI (continuous z-score), and smoke exposure (yes/no), which were selected according to the literature. We decided to use a weighted quantile sum (WQS) approach in our analyses to examine the mixture effect and to identify the components associated with a health outcome, as well as to account for autocorrelations among the data. This approach was used in two ways: (1) to assess cardiovascular risk with a multi-OxS biomarker mixture and (2) to assess individual OxS biomarkers with a urine metal(loid) mixture. Individual OxS biomarker predictors were divided into quartiles to assess the multi-metal(loid) mixture, and the same was done with the individual OxS biomarkers to assess cardiovascular risk. Our final WQS model included weights that were the mean weight across 100 bootstrapped datasets and constrained to be both non-negative. All models were adjusted for child age, sex, and waist-to-height ratio (WHtR). We normalized metal(oid) concentration to urinary density. Analyses were conducted using the statistical package STATA 13.0 (StataCorp. LP) and R software Version 4.3.2. A *p*-value ≤ 0.05 was considered statistically significant. The “Statistical Analysis Tools” toolbox of the ArcGIS 10.5 geographic information system was used to calculate spatial autocorrelation and estimate hot spots (Gi* from Getis-Ord.). For statistically significant positive z-scores, the clustering of high values is stronger for hot spots. For statistically significant negative scores, the clustering of low values is stronger for cold spots.

## Results

A total of 359 patients were evaluated, 54.4% of which were women. The population’s median age was 14 years and ranged between 12 and 18 years. We found that about 30% of the population was overweight or obese according to the BMI calculated with the methodology of the CDC for pediatric populations. However, when using the waist-to-height ratio (WtHr), approximately 40% of the population exceeded the cut-off point of 0.5, which indicates overweight/obesity. In the case of glomerular filtration rate (eGFR), 3.1% of the participants exhibited values below 70 mL/min/m^2^ (which corresponds to a severe decrease in GFR in a pediatric population), 23.7% showed a slight decrease (71 to 89 mL/min/m^2^), 1.9% showed hyperfiltration (eGFR > 150 89 mL/min/m^2^), and 71.3% exhibited normal eGFR values. Furthermore, 19% of the population (including both sexes) stated that they were smokers. Finally, physical activity was also quantified and classified as low or null in 23.1% of the population (if they exercised one day a week or not at all), moderate in 57.7% (if they exercised two to four days per week), and high in 19.2% (four or more days of exercise per week) (Table 1).

**Table 1:** General characteristics of the population and exposure to the biomarkers evaluated

Variable	All (359)
*Sex, n (%)* Male Female	163 (45.4) 196 (54.6)
*Age, years (median, IQR)*	14 (13–16)
*WtHR (median, IQR)*	0.482 (0.443–0.528)
*BMI, n (%)* Normal weight Overweight Obesity	250 (69.6) 71 (19.8) 38 (10.6)
*eGFR, n (%)* < 70 mL/min/m^2^ 70 to 89 mL/min/m^2^ 90 to 150 mL/min/m^2^ > 150 mL/min/m^2^	11 (3.1) 85 (23.7) 256 (71.3) 7 (1.9)
**Habits**	
*Smoker, n (%)* Yes No	19 (5.3) 340 (94.7)
*Physical activity, n (%)* Low or null Moderate High	83 (23.1) 207 (57.7) 69 (19.2)
***Cardiovascular risk n (%)*** Yes No	117 (32.6) 242 (67.4)
**Plasma oxidative stress biomarkers** (median, IQR)	
AOPPs mmol/g	1.6 (0.98–3.04)
*ARG* µg urea/mg	0.52 (0.4–0.67)
*CAT* U/g	1.56 (1.06–2.25)
*GGT* U/g	0.18 (0.12–0.26)
*GST* pmol/min/mg	0.23 (0.16–0.3)
*MDA* µM/mg	3.25 (1.8–6.02)
*MGO* µM/mg	0.73 (0.37–1.58)
*MPO* U/g	58.03 (37.3–97.3)
*NEFAs* µM/mg	1.97 (1.08–4.14)
**Urine metal(loid) concentrations** (median, IQR)	
Arsenic ng/mL	34.549 (25.227–46.169)
Copper ng/mL	27.255 (19.231–50.768)
Manganese ng/mL	0.837 (0.014–2.245)
Vanadium ng/mL	7.4514 (2.959–13.92)

Abbreviations: *BMI* Body mass index, *WtHR* Waist to height ratio, *eGFR* Estimated glomerular filtration rate, *AOPPs* Advanced oxidation protein products, *ARG* Arginase, *CAT* Catalase, *GGT* Gamma-glutamyl transferase, *GSTs* Glutathione-S-Transferases, *MDA* Malondialdehyde, *MGO* Methylglyoxal, *MPO* Myeloperoxidase, *NEFAs* Non-esterified fatty acids, *IQR* Interquartile range

Cardiovascular risk was identified in 32.6% of the population. To determine cardiovascular risk, we used the recommendations of a panel of experts that integrates different guidelines (Expert Panel on Integrated Guidelines for Cardiovascular et al., 2011) for managing cardiovascular health in adolescents (Table 2).

**Table 2:** Stratification of cardiovascular risk factors in the population

Category	Conditions
Cardiovascular risk	Present at least 3 risk factors (2 factors in case of extreme values) • Diabetes mellitus • Kidney disease (eGFR < 70 mL/min/m^2^) • Blood pressure^a^ > 90th percentile • BMI^a^ > 85th percentile • Walking or light exercise less than 2 times per week • Smoking
No apparent cardiovascular risk	If they only present one of the aforementioned factors or… • eGFR > 70 mL/min/m^2^ • Blood pressure^a^ < 90th percentile • BMI^a^ < 85th percentile • Light to vigorous exercise more than 2 times per week • No smoking history

Abbreviations: *BMI* Body mass index, *eGFR* Estimated glomerular filtration rate

ª Variable adjusted for age and sex in the pediatric population

Table 1 shows the concentrations and enzymatic activities of the OxS biomarkers, including AOPPs, ARG, CAT, GGT, GST, MDA, MGO, MPO, and NEFAs, normalized to protein concentration. The metal(oid)s determined in the participants’ urine samples were: As (median: 34.5 ng/mL), Cu (median: 27.2 ng/mL), Mn (median: 0.837 ng/mL), and V (median: 7.45 ng/mL). Other elements, such as mercury, cadmium, or chromium, were analyzed but detected in less than 10% of the population or were entirely undetectable (data not shown).

Table 3 shows the correlations of the OxS biomarkers with the metal(loid)s. Positive and significant correlations were observed with AOPPs, GST, MGO, and NEFAs, and marginal correlations with MPO. Copper showed a marginal correlation with GST. Manganese correlated positively with CAT, but negatively with MDA and NEFAs, and showed a marginal correlation with ARG. Finally, V showed a significant correlation with NEFAs and GST. The only biomarker that did not show any correlation, significant or marginal, with any metal(loid) was GGT.

**Table 3:** Correlations between oxidative stress biomarkers and urine metal(loid)s in the population studied

Variable	AOPPs	ARG	CAT	GGT	GST	MDA	MGO	MPO	NEFAs
As	0.146*	0.026	− 0.026	0.026	0.114*	− 0.054	0.138*	0.098ª	0.123*
Cu	− 0.024	0.061	0.024	0.086	0.091ª	0.011	0.015	0.016	0.011
Mn	− 0.039	0.102ª	0.147*	0.016	− 0.001	− 0.145*	0.063	− 0.046	− 0.105*
V	0.053	0.037	− 0.018	0.009	0.141*	0.039	0.004	0.025	0.134*

Abbreviations: *AOPPs* Advanced oxidation protein products, *ARG* Arginase, *CAT* Catalase, *GGT* Gamma-glutamyl transferase, *GSTs* Glutathione-S-Transferases, *MDA* Malondialdehyde, *MGO* Methylglyoxal, *MPO* myeloperoxidase, *NEFAs* Non-esterified fatty acids. As Arsenic, Cu Copper, Mn Manganese, V Vanadium. Spearman’s correlation; ª *p* > 0.05 to *p* < 0.1, * *p* < 0.05

The results of the logistic regression of cardiovascular risk, individual OxS biomarkers, and individual metal(loid)s are shown in Table 4. No significant or marginal associations were observed between any urine metal(loid) and cardiovascular risk. However, in the case of individual OxS biomarkers, there were significant associations between cardiovascular risk and OAPPs, MGO, and MPO. OAPPs were associated with an increase of 0.1337 ng/mL (95% Confidence Interval (CI): 0.0219–0.2456) in individuals with cardiovascular risk; MGO was associated with an increase of 0.2187 ng/mL (95% CI: 0.0514–0.3859) in individuals with cardiovascular risk; and MPO was associated with an increase of 0.0.0049 ng/mL (95% CI: 0.0012–0.0085) in individuals with cardiovascular risk.

**Table 4:** Logistic regression of cardiovascular risk and oxidative stress biomarkers and metal(oid)s

OxS biomarker	Beta	95% CI	Metal(oid)	Beta	95% CI
AOPPs	0.134	0.0219–0.2456 *	As	-0.0008	− 0.009–0.007
ARG	0.232	− 0.1203–0.584	Cu	0.001	− 0.0023–0.004
CAT	0.085	− 0.0807–0.2498	Mn	0.019	− 0.014–0.052
GGT	− 0.96	− 2.3167–0.3868	V	0.048	− 0.198–0.295
GST	− 1.56	− 3.4299–0.317			
MDA	0.011	− 0.0106–0.0318			
MGO	0.219	0.0514–0.3859 *			
MPO	0.005	0.0012–0.0085 *			
NEFAs	− 0.011	− 0.0752–0.0522			

Abbreviations: *AOPPs* Advanced oxidation protein products, *ARG* Arginase, *CAT* Catalase, *GGT* Gamma-glutamyl transferase, *GSTs* Glutathione-S-Transferases, *MDA* Malondialdehyde, *MGO* Methylglyoxal, *MPO* Myeloperoxidase, *NEFAs* Non-esterified fatty acids, *CI* Confidence interval; ^*^ *p* < 0.05

**Fig. 1:**
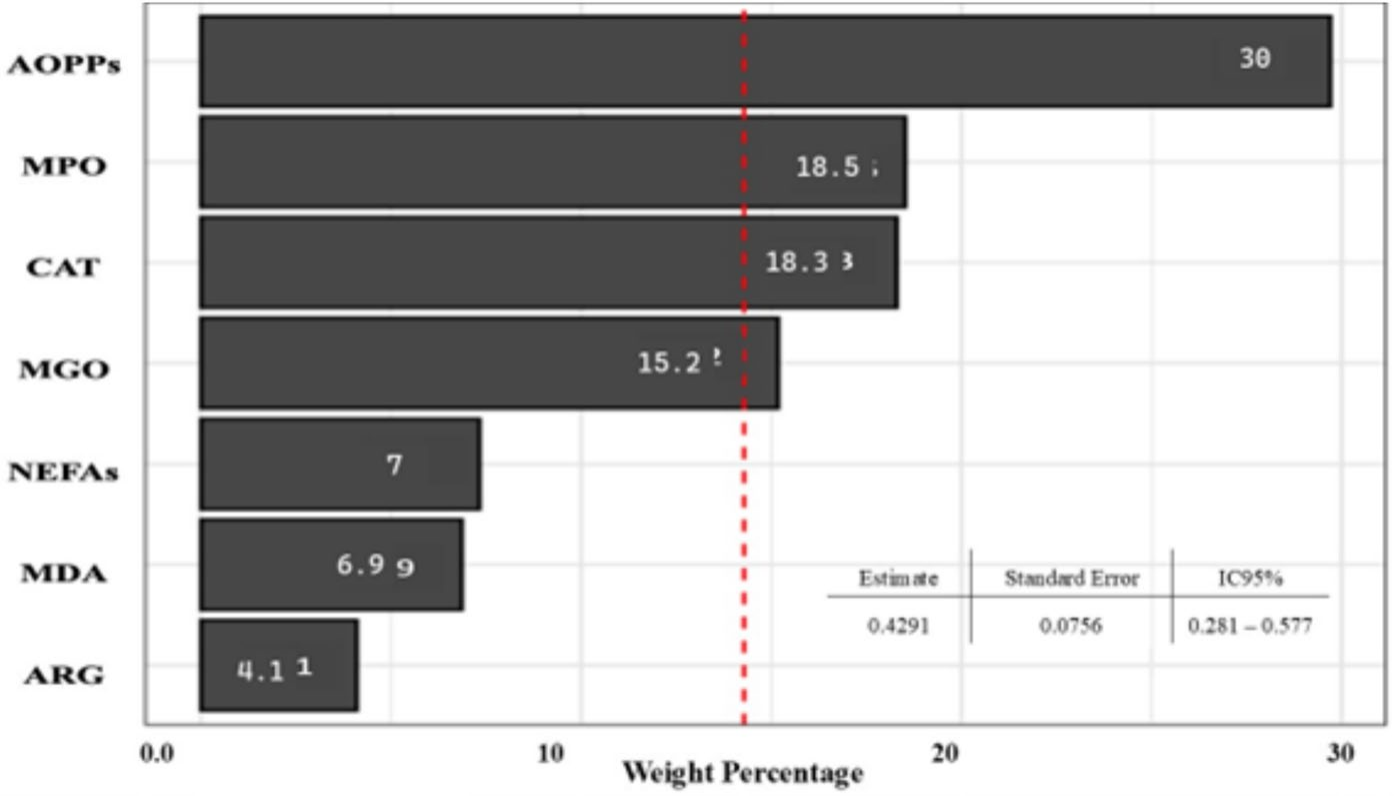
WQS regression model weights of the contribution of each oxidative stress biomarker to the overall effect. We used a WQS regression to analyze the relationship between plasma oxidative stress biomarker co-exposure and cardiovascular risk in a positive direction. The estimate was adjusted for age and sex. Abbreviations: *WQS* weighted quantile sum, *AOPPs* Advanced oxidation protein products, *ARG* Arginase, *CAT* Catalase, *GGT* Gamma-glutamyl transferase, *GSTs* Glutathione-S-Transferases, *MDA* Malondialdehyde, *MGO* Methylglyoxal, *MPO* myeloperoxidase, *NEFAs* Non-esterified fatty acids

The results of the individual associations between cardiovascular risk and the multi-OxS biomarker mixture are shown in Fig. 1. The WQS constrained in both the positive and negative direction resulted in positive beta estimates; therefore, we constrained the WQS in the positive direction and present those results. The multi-OxS biomarker indices were associated with the population considered to be at cardiovascular risk. For each quartile increase in the multi-OxS biomarker mixture, there was an increase in the odds of being at cardiovascular risk (OR = 0.4291,95% CI: 0.281–0.577). The contributions of the OxS biomarkers associated with increased cardiovascular risk levels were, from higher to lower, as follows: AOPPs (30%), MPO (18.5%), CAT (18.3%), and MGO (15.2%). Even though NEFAs, MDA, and ARG also contributed to the association, their contribution was limited. The WQS estimates for the association of the multi-metal(loid) mixture with cardiovascular risk did not show any significant results. However, there were significant correlations between the OxS biomarkers and the metal(loid)s detected in the urine. Hence, we looked at the association between the urine metal(loid) weights derived for the multi-metal(loid) index in the WQS and each OxS biomarker.

The results of the WQS regression analysis of the associations between the urine metal(loid) mixture and the individual OxS biomarkers are shown in Table 5. We found that for each quartile increase in the urine metal(loid) mixture, there was an increase of 0.0492 ng/mL in log-AOPPs (95% CI: 0.0175–0.081), which was attributable to the contribution of As (55.5%), V (25.5%), Cu (11.7%), and Mn (7.3%). For each quartile increase in the urine metal(loid) mixture, there was an increase of 0.036 ng/mL in log-ARG (95% CI: 0.0077–0.064), which resulted from the contributions of Mn (41.9%), V (32.7%), Cu (18.1%), and As (7.3%). For each quartile increase in the urine metal(loid) mixture, there was an increase of 0.1073 ng/mL in log-MGO (95% CI: 0.0759–0.139) attributable to the contributions of As (49.1%), Mn (25.5%), Cu (12.9%), and V (12.4%). Finally, for each quartile increase in the urine metal(loid) mixture, there was an increase of 0.0313 ng/mL in log-MPO (95% CI: 0.0119–0.051) resulting from the contributions of As (65.7%), V (16.2%), Cu (13.3%), and Mn (4.8%).

**Table 5:** Associations between individual oxidative stress biomarkers and the multi-metal(loid) mixture and the derived metal(loid) weights

	Estimate	Standard error	IC 95%	Metal(loid) weights (%)			
				W1	W2	W3	W4
log-AOPPs	0.0492	0.016	**0.0175–0.081**	As: 55.5	V: 25.5	Cu: 11.7	Mn: 7.3
log-ARG	0.036	0.0144	**0.0077–0.064**	Mn: 41.9	V: 32.7	Cu: 18.1	As: 7.3
log-CAT	0.0091	0.0121	− 0.015–0.033	Mn: 57	As: 20.8	Cu: 17.2	V: 5
log-MDA	-0.014	0.0365	− 0.0867–0.058	V: 48.3	Cu: 44.1	As: 4.4	Mn: 3.2
log-MGO	0.1073	0.016	**0.0759–0.139**	As: 49.1	Mn: 25.5	Cu: 12.9	V: 12.4
log-MPO	0.0313	0.0099	**0.0119–0.051**	As: 65.7	V: 16.2	Cu: 13.3	Mn: 4.8
log-NEFAs	0.0018	0.0295	− 0.056–0.06	Cu: 43.2	As: 40.9	V: 13.2	Mn: 2.7

Models shown were constrained in the positive direction with 100 repeated holdout validations, adjusted for child age and sex. Abbreviations: *AOPPs* Advanced oxidation protein products, *ARG* Arginase, *CAT* Catalase, *GGT* Gamma-glutamyl transferase, *GSTs* Glutathione-S-Transferases, *MDA* Malondialdehyde, *MGO* Methylglyoxal, *MPO* Myeloperoxidase, *NEFAs* Non-esterified fatty acids, As Arsenic, Cu Copper, Mn Manganese, V Vanadium. Bold values indicate statistical significance (*p*<0.05)

**Fig. 2:**
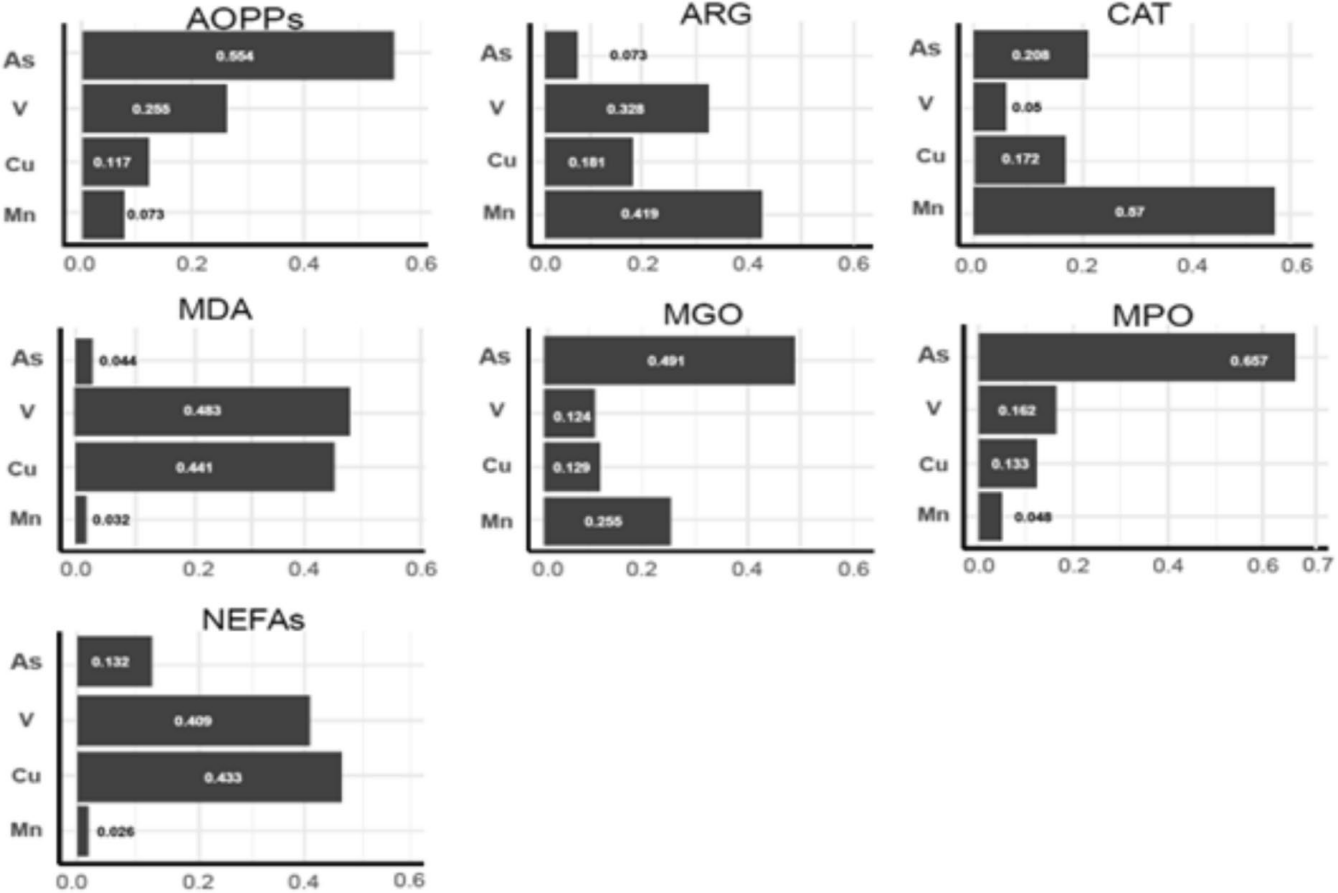
Oxidative stress biomarker weights derived for the multi-metal(loid) index in weighted quantile sum regression analyses with individual plasma biomarkers. For *AOPPs* Advanced oxidation protein products, *ARG* Arginase, *CAT* Catalase, *MDA* Malondialdehyde, *MGO* Methylglyoxal, *MPO* Myeloperoxidase, and *NEFAs* Non-esterified fatty acid. Models shown were constrained in the positive direction with 100 repeated holdout validations, adjusted for child age, sex, and waist-to-height ratio

There were no significant associations between log-CAT, log-MDA, log-NEFAs, log-GGT, and log-GST and any of the urine metal(loid) mixture indices. The urine metal(loid) weights derived for the multi-metal(loid) index in the WQS with each OxS biomarker are shown in Fig. 2.

The results of the spatial autocorrelation analysis showed a generally low spatial correlation. Two tests were conducted with distance bands of 1100 m and 1800 m. These values were obtained using an incremental spatial autocorrelation analysis and a hot spot analysis (Getis-Ord Gi*). In both cases, the hot spot cluster areas were very similar. Of the four metal(loid)s analyzed, statistically significant differences were observed in Global Moran’s I for vanadium and arsenic.

**Fig. 3:**
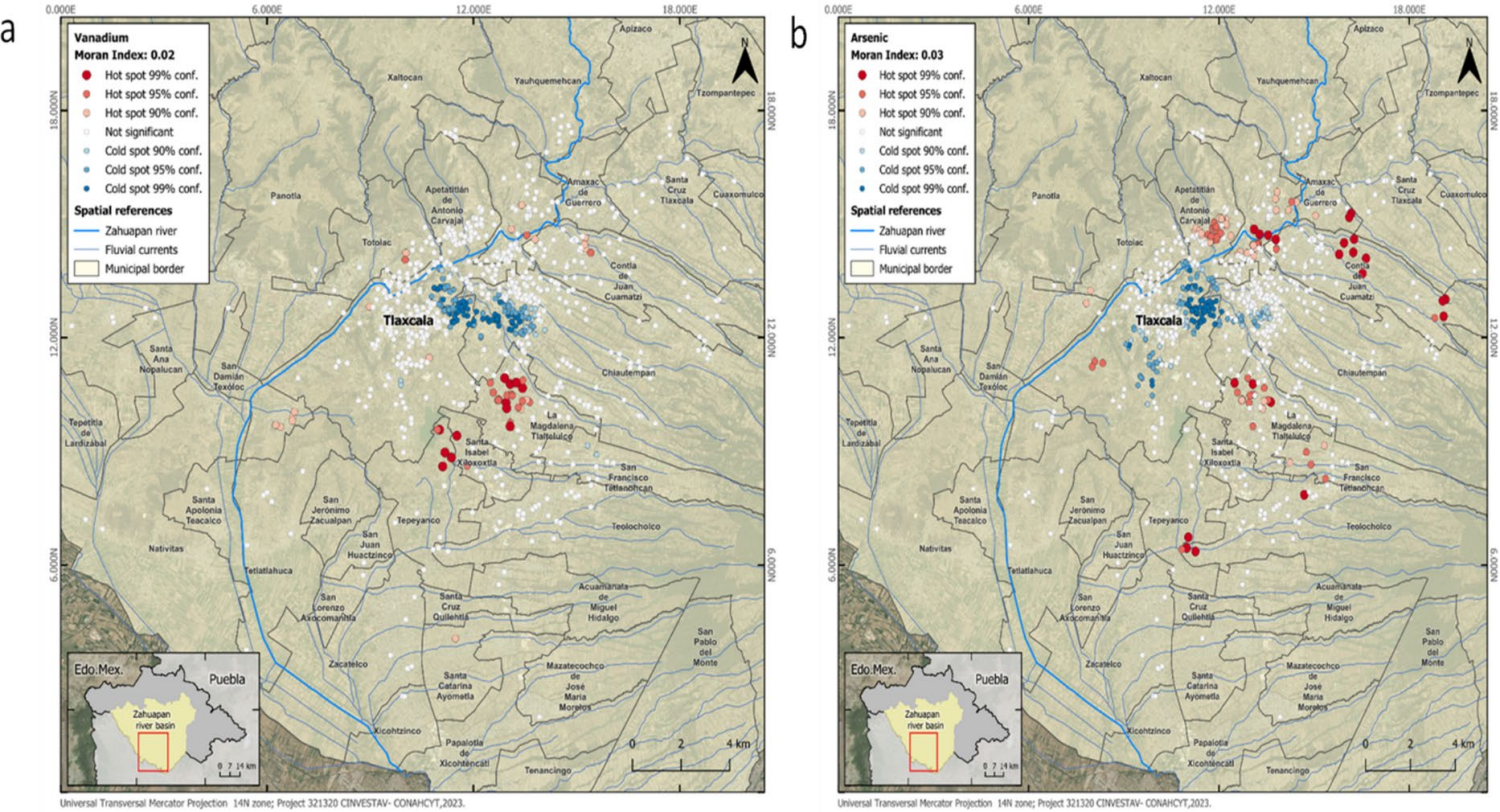
Hot spot maps of the spatial autocorrelation between industrial sources and urinary metal(loid) concentrations in pediatric participants. **a** Spatial autocorrelation of vanadium and **b** Arsenic

In the case of Vanadium, the results showed a Global Moran’s I value of 0.02 and a Z value of 2.69, indicating regional clustering of the data. The areas where hot spots were observed were analyzed based on industrial proximity (Fig. 3a), which was within 1 km of the subjects. Standout small companies were engaged in the manufacture of artisanal wood products, artisanal food, artisanal recycled threads and textiles, concrete, bricks, block makers, and metal products. Large companies, which were found within 1 km of each other, were engaged in the manufacture of textiles, non-metallic products, and ceramic products.

The resutls for arsenic showed a Global Moran’s I value of 0.03 and a Z value of 5.5, indicating regional clustering of the data. Small companies located within 1 km of the hot spots were mainly engaged in the production of textiles, artisanal wood, artisanal food, concrete pipes, and metal products. Larger companies were engaged in the manufacturing and dyeing of textiles and the manufacturing of bags and plastic products and ceramic products (Fig. 3b).

**Fig. 4:**
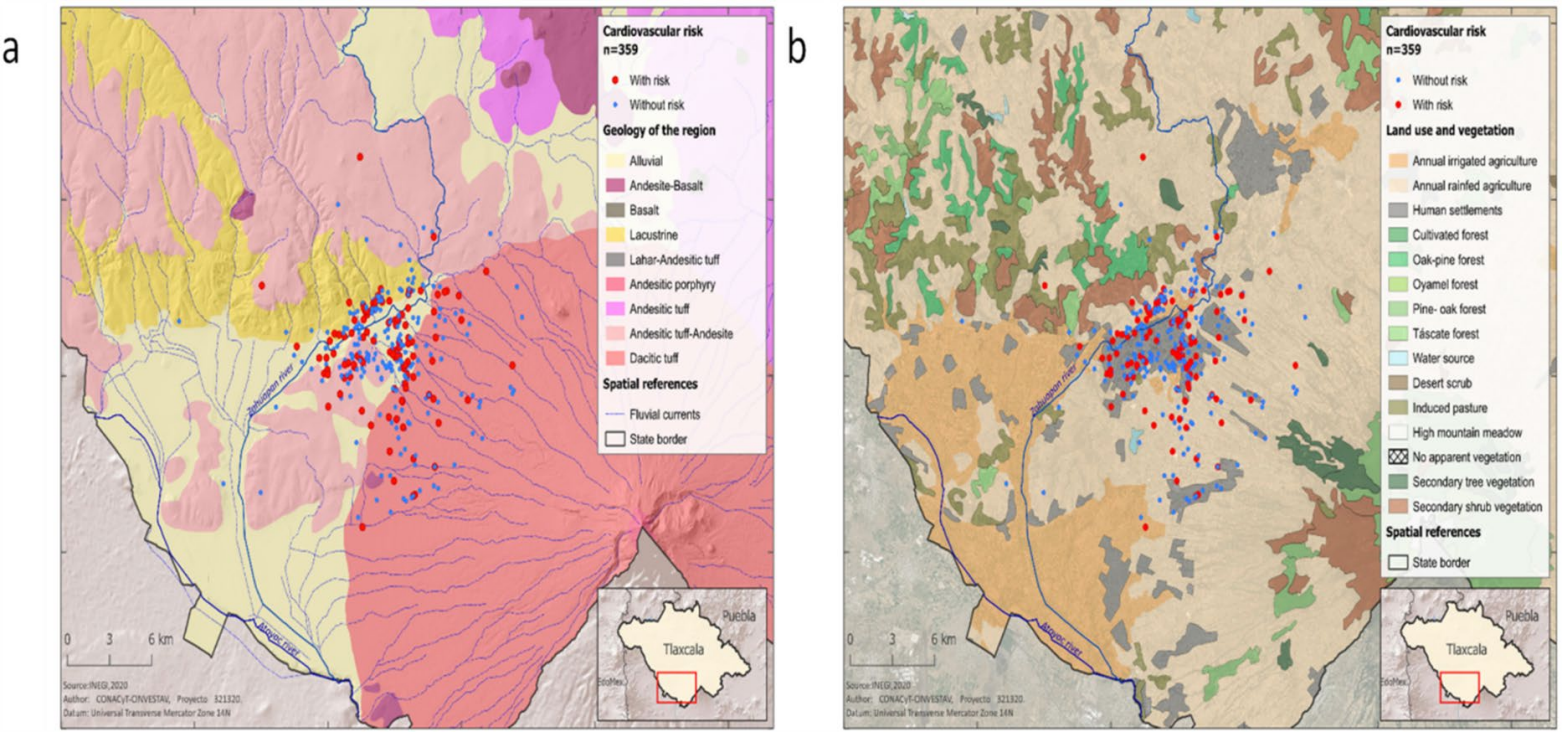
Maps of the spatial distribution of the pediatric population studied based on the geology and land use in the region. **a** Geology of the region, and **b** Land use and vegetation in the region

In addition to these findings, vanadium and arsenic are naturally present in the soil of human settlementsthat are located on volcanic substrates near the dormant Malinche Volcano (Fig. 4a), within or near which the pediatric population studied was geolocated. In these areas, various economic, industrial, and agricultural activities may contribute to soil erosion and its dispersion through the air (Fig. 4b).

## Discussion

Oxidative balance is a process that can be altered by various factors, including environmental pollutants such as inorganic elements, toxic metals and metalloids. There is evidence that toxic elements can interact with DNA and proteins, causing oxidative deterioration of biological macromolecules. The mechanisms by which they can cause these effects are varied, but OxS remains one of the most studied (Bertini & Cavallaro, 2008; Valko et al., 2005). Since the generation of free radicals in living systems is closely linked to the participation of redox-active metals such as iron, copper, chromium, and cobalt, the cell’s redox state is maintained within strict physiological limits (Jomova & Valko, 2011).

OxS is a biological process that has been defined as the lack of balance between the occurrence of reactive oxygen/nitrogen species (ROS/RNS) and the organism’s capacity to counteract their action by antioxidant protection systems and mechanisms and endogenous and exogenous molecules (Persson et al., 2014). It has been established that OxS, which favors oxidants, leads to damage to all essential bio-compounds, such as proteins, DNA, and membrane lipids, and can lead to cell death (Pisoschi & Pop, 2015). As a result, OxS has been confirmed to contribute to many chronic health problems such as emphysema and inflammatory and cardiovascular diseases (Gutteridge, 1993; Halliwell et al., 1992; Lopez-Alarcon & Denicola, 2013).

To assess cardiovascular risk (CVR), we followed the recommendations for the pediatric population (Expert Panel on Integrated Guidelines for Cardiovascular et al., 2011), since there are differences with the values recommended for the adult population. Based on the guidelines for cardiovascular health, we observed that more than 30% of the pediatric population met the criteria for CVR. Hence, if the pediatric population does not make any lifestyle changes or eliminate the risk factors, CVR could turn into cardiovascular disease over time, with an assumed prevalence that could reach 30%, similarly to a cohort of 7-year-old Colombian children that showed CVR close to 40% (Briceno et al., 2015).

We observed a median urinary arsenic concentration of 34.549 ng/mL, which exceeds the reference value considered normal (20 ng/mL) (Hays et al., 2010) and is high in the open population. High arsenic concentrations have been related to various diseases in several population studies, with an increased incidence of ischemic heart disease and cardiovascular mortality (Stea et al., 2014). The median Cu urine concentration was 27.255 ng/mL. This metal is a cofactor of several enzymes and is associated with important biological processes including angiogenesis, response to hypoxia, and neuromodulation (Scheiber et al., 2013). However, excess intracellular free copper ions are potentially toxic, since they are related to the generation of reactive oxygen species (ROS) (Chen et al., 2020). Manganese is an essential dietary element that functions primarily as a coenzyme in biological processes such as macronutrient metabolism, bone formation, free radical defense systems, and brain processes (Erikson & Aschner, 2019). Overexposure to environmental Mn can induce parkinsonism, although there is limited information on how Mn can induce cardiovascular dysfunction (Jiang & Zheng, 2005). Finally, vanadium showed a median concentration of 7.4 ng/mL, which is below the reference value (10 ng/mL) (Nordberg and Costa, 2021; Nordberg et al., 2022). Elevated concentrations of vanadium in the adult population have been associated with mortality due to cardiovascular accidents (Clark et al., 2014; Shi et al., 2021).

The metal(loid)s found in the present study did not show an association with cardiovascular risk, but they showed significant correlations with the OxS biomarkers analyzed (AOPPs, ARG, CAT, GGT, GST, MDA, MGO, MPO, and NEFAs). These results are related to alterations in the homeostasis of metal(loid)s in the body, including the dysregulation of uptake, storage, and excretion mechanisms; these toxic metal(loid)s can cause oxidative damage to biological macromolecules, thereby interfering with various biological processes (Jomova & Valko, 2011).

In the population not occupationally exposed to metal(loid)s, exposure to these xenobiotics occurs through contaminated water, soils, air, or foods, and an important aspect is that it is not only one but a mixture. Our results showed that the metal(loid) mixture was significantly associated with changes in AOPPs, ARG, MGO, and MPO, with each element contributing with a different weight to the change in each OxS biomarker. Even though As and V were observed to have the greatest contribution to the changes in AOPPs, MGO, and MPO, Cu and Mn cannot not be discarded because all these elements interact within the body.

As and V are elements considered unnecessary for the body, just as cadmium or lead, since they have no known biological function and are even known to be toxic at low concentrations. The presence of As has been related to endothelial dysfunction (Wu et al., 2012) and, when combined with smoking, to ischemic heart disease (Chen et al., 2011), among other conditions. Vanadium is a toxic metal and a potent environmental and occupational pollutant that can cause neurotoxicity, testicular toxicity, and kidney damage (Zwolak, 2020), although the results of different studies are ambiguous (Ghalichi et al., 2022). In our study, neither As nor V showed a significant association with cardiovascular risk, but were significantly related to the OxS biomarkers evaluated.

The primary route of the relationship of both of these elements with OxS is the depletion of glutathione, bonding to sulphydryl groups of proteins and other mechanisms, which is consistent with the significant associations of both elements with AOPPs, ARG, MGO, and MPO observed in our study (Sinicropi et al., 2010). When organisms are exposed to As, they undergo the formation of peroxyl radicals (ROO·), superoxide, singlet oxygen, hydroxyl radical (OH·), and hydrogen peroxide, which also induce oxidized lipids that generate other molecules like peroxides, isoprostanes, and mostly MDA and 4-hydroxy-nonenal (Jomova et al., 2011). This indicates that the presence of As is involved in an increase in the concentrations of MPO, MGO, ARG, and AOPPs. Vanadium is associated with the production of reactive oxygen species, which leads to lipid peroxidation and changes in antioxidant enzymes, and has also been observed to accumulate in the mitochondria, resulting in increases in catalase and superoxide dismutase activities (Soares et al., 2008).

On the other hand, Cu and Mn are elements considered biologically active. For example, redox-active metals such as Cu may undergo cycling reactions of transfer of electrons between metals and substrates, whose disruption may lead to an uncontrolled metal-mediated formation of deleterious free radicals (Valko et al., 2007), or, paradoxically, are an integral part of active sites of antioxidant enzymes that battle the toxic effects of metal-induced free radicals (Fukai & Ushio-Fukai, 2011). OxS associated with Cu is partly a consequence of its redox reactivity, which is the ability of free Cu or low molecular weight Cu complexes to catalyze the reaction between superoxide anion and H_2_O_2_, thereby producing the hydroxyl radical. In addition, Cu can bind directly to free thiols of cysteines, which can result in oxidation and subsequent crosslinks between proteins, leading to impaired activity (Cecconi et al., 2002).

Each element in the mixture contributed in different proportions to these changes, where significant contributions were found in the case of AOPPs, ARG, MGO, and MPO. Given that these biomarkers of OxS showed a significant association with cardiovascular damage, it is possible to suggest that metal(loid)s alter the redox balance in the body, which results in apparent cardiovascular damage.

In the present study, we quantified a mixture of different OxS biomarkers, which were individually significantly associated with vascular damage, mainly AOPPs, MGO, and MPO. In order to determine whether the presence of and alterations in various OxS biomarkers influence cardiovascular damage, we applied a WQS regression model, which showed that, in addition to the three biomarkers mentioned above, catalase is also significantly related to an increase in cardiovascular damage; NEFAs, MDA, and ARG showed a lower contribution to an increase in cardiovascular damage and were not statistically significant; finally, GGT and GST did not appear to contribute at all to an increase in cardiovascular damage. Cardiovascular alterations or damage have been previously related to an imbalance in the redox state of the organism, both in animal models and human populations, where there has been a relationship with the same biomarkers of OxS used in our study or similar ones, and has been related to processes such as inflammation, diabetes, dyslipidemia, hypertension, atherosclerosis, etc., or directly to the malfunction of enzymes of the antioxidant system such as SOD, MPO, NADPH, among others (Pignatelli et al., 2018; Steven et al., 2019).

In our study, of the multiple OxS biomarkers evaluated, AOPPs appeared to be the most representative biomarker in individuals with cardiovascular risk, with approximately 30% of weight in the mixture. This result may be explained by some of the characteristics that have been reported for AOPPs. AOPPs can form under many conditions, including oxidant-antioxidant imbalance, glycol oxidation processes, and coexisting inflammation; however, most AOPPs are the result of myeloperoxidase-derived OxS (Ou et al., 2017). AOPPs are structurally analogous to advanced glycation end products (AGEs) with the ability to induce pro-inflammatory cytokines. High levels of AOPPs have been related to diabetes, cardiovascular diseases, hypertension, and atherosclerosis (Conti et al., 2019).

Studies have shown that hypercholesterolemia can spontaneously produce AOPPs, suggesting that hyperlipidemia may enhance the in vivo process of AOPP formation by increasing OxS. The formation of AOPPs is irreversible and they cannot be easily hydrolyzed by proteolytic enzymes or reduced by antioxidants such as vitamin C and glutathione (Ou et al., 2017). AOPPs accumulate with age and catalyze structural and functional shifts within the cardiovascular system, including vascular stiffening, reduced arterial compliance, myocardial abnormalities, and endothelial dysfunction. These processes are often exacerbated by comorbidities (Rasool et al., 2019). Beyond traditional risk factors, chronic inflammation serves as a primary driver of cardiovascular disease (CVD) by inducing deleterious changes in lipid profiles, specifically affecting LDL, HDL, and triglycerides. Pro-inflammatory and oxidative stress-linked enzymes, such as myeloperoxidase (MPO), can modify the structural integrity of these lipoproteins. Notably, oxidized HDL (oxHDL) is prevalent in atherosclerotic plaques and is strongly associated with heightened coronary artery disease risk (Ungurianu et al., 2017).

These mechanisms underscore why AOPPs emerged as the most significant oxidative stress biomarker in our cardiovascular risk assessment, particularly when adjusted for variables such as weight, renal health, and blood pressure. While the present study did not measure lipid parameters (LDL, HDL, or TGs) to confirm these specific metabolic pathways in our cohort, our findings identify MPO and MGO as additional significant contributors to cardiovascular risk. This aligns with existing literature reporting elevated MPO and MGO concentrations in high-risk populations as indicators of poor prognosis and impaired recovery (Mahat et al., 2019; Ramachandra et al., 2020).

MPO is implicated in the pathogenesis of multiple inflammatory diseases, including atherosclerosis and cardiovascular disease (Aratani, 2018). High concentrations of MPO have been observed to increase cardiovascular risk two- to tenfold (Anatoliotakis et al., 2013; Schindhelm et al., 2009). MPO is involved in hypertension, diabetes, triglyceride and fatty acid metabolism, etc. (Meuwese et al., 2007; Ramachandra et al., 2020). In a subpopulation of the European Prospective Investigation Into Cancer and Nutrition-Norfolk study, elevated serum concentrations of MPO were associated with an increased risk of cardiovascular disease. An increase in inflammatory biomarkers was observed to precede the onset of cardiovascular disease as age increased (Ndrepepa, 2019) in a population with participants of approximately 65 years. However, the population in our study was under the age of 18. The close relationship between MPO and AOPPs, where AOPPs are part of the oxidative metabolism of MPO, explains why MPO is so prevalent in individuals considered to have cardiovascular damage.

MGO is a highly reactive dicarbonyl compound, the primary precursor for non-enzymatic glycation, which leads to advanced glycation end-products (AGEs), and has been implicated in type 2 diabetes, cardiovascular disease, cancer, and nervous system disorders (Schalkwijk & Stehouwer, 2020). High concentrations of MGO in obese individuals may be explained by alterations in the metabolism of proteins, nucleic acids, glycolysis, and lipid peroxidation, which give rise to highly reactive molecules such as MGO (Hernandez-Castillo & Shuck, 2021; Matafome et al., 2013). MGO also causes endothelial and vascular dysfunction, OxS, and atherosclerosis, which are all risk factors for cardiovascular disease (Sankaralingam et al., 2018). Considering that the Mexican population is one of the most affected by metabolic disorders, the presence of MGO in our participants could be considered an important biomarker of cardiovascular risk.

Finally, catalase was another OxS biomarker with more weight in individuals with cardiovascular risk. Catalase is a tetrameric heme protein that catalyzes the disproportionation of H_2_O_2_ to water and oxygen, and can detoxify hydrogen peroxide into water at higher H_2_O_2_ concentrations because of a decrease in the activity of other enzymes, which explains a higher activity of CAT and could be a compensatory response to counteract OxS and may serve as an adaptive response to elevated levels of ROS (Bizon et al., 2023). Various diseases involving OxS have been observed to be accompanied by an increase in catalase activity, including cardiovascular diseases, diabetes, tumors, infections, and inflammations, among others (Al-Abrash et al., 2000).

While hydrogen peroxide is mainly formed by superoxide dismutases, it is rapidly degraded by catalase and glutathione peroxidase. Catalase in the endothelium is associated with a reduction in systolic blood pressure in in vivo models and is weakly protective against myocardial ischemia–reperfusion injury (Suvorava & Kojda, 2009). Low catalase expression levels or activity may result in an incrase in the concentration of hydrogen peroxide in the cells, causing OxS, mutagenesis, and inflammation; low catalase activities have also been reported in schizophrenic patients and patients with atherosclerosis (Nandi et al., 2019). In this study, we observed alterations in different biomarkers of OxS, and thus the enzymatic activity of the antioxidant system would also be altered, and although we did not evaluate SOD, it is psosible to assume that it would be altered as well. Catalase usually works more efficiently in conjunction with other enzymes of the same system, so it is not surprising that we found that it is a biomarker that apparently contributes to cardiovascular risk.

In the case of the geolocation analysis, the data suggest that the main sources associated with arsenic and vanadium exposure are the textile, metalworking, and ceramics industries. Although these industrial activities do not use these chemicals in the manufacture of their products, they may contribute to exposure to these metal(loid)s by promoting soil erosion and dust resuspension throughthe transportation of raw materials and the deforestation of areas where they operate. It should be noted that the soil layer may contribute significantly to the presence of metal(loid)s (i.e. alumminium, arsenic, silice, among others) given that the state of Tlaxcala is located in a volcanic region. Therefore, deforestation and soil erosion, as well as agricultural and construction activities, may be contributing to children’s exposure through airborne routes. The composition of volcanic soil can contribute to concentrations of both vanadium and arsenic, as has been demonstrated at other sites in Mexico and elsewhere around the world that are of volcanic geological origin or located near volcanic areas (Hernandez & Rodriguez, 2012; Vargas-Solano et al., 2019).

In conclusion, OxS biomarkers were associated with cardiovascular risk and some related factors. Specifically, the presence of metal(loid)s—known to drive redox imbalances—appears to modulate the behavior of these biomarkers, thereby escalating cardiovascular susceptibility. Our findings suggest that AOPPs, MPO, MGO, and CAT may serve as robust indicators of cardiovascular risk in populations subjected to environmental pollutants, particularly metal(loid)s. Nevertheless, further large-scale longitudinal studies, particularly those incorporating pediatric cohorts with existing comorbidities or exposure to diverse xenobiotics, are required to validate these biomarkers. Such research is essential to fully elucidate the diagnostic utility and mechanistic relationship between OxS biomarkers and cardiovascular pathology. Finally, the relation between exposure to vanadium and arsenic and the presence of industrial activities, and perhaps the environmental contribution of the volcanic soil where the population is located, should be considered thoroughly.

We recognize that our study is subject to different limitations. For instance, potential sources of exposure to metal(loid)s were not identified at all, and there was no information available to estimate the influence of the diet of the participants. Therefore, we cannot exclude the possibility of exposure to other environmental toxicants that could have interfered with the observed results. Genetic characteristics and dietary supplementation were not included in the study. As a cross-sectional study, the concern of reverse causation cannot be ignored, and no causation could be established. Thus, the causal relationship between metal exposure and cardiovascular risk needs further investigation, such as prospective cohort studies or studies with a stratified analysis under a larger sample size.

## Data Availability

The datasets used and analyzed during the current study are available from the corresponding author on reasonable request.
